# Poverty related risk for potentially preventable hospitalisations among children in Taiwan

**DOI:** 10.1186/1472-6963-10-196

**Published:** 2010-07-07

**Authors:** Likwang Chen, Hsin-Ming Lu, Shu-Fang Shih, Ken N Kuo, Chi-Liang Chen, Lynn Chu Huang

**Affiliations:** 1 Division of Health Policy Research and Development, The Institute of Population Health Sciences, National Health Research Institutes, Taiwan; 2 Institute of Public Health, School of Medicine, National Yang-Ming University, Taiwan; 3 The Department of Accounting, The College of Business, Chung Yuan Christian University, Taiwan

## Abstract

**Background:**

This study investigated the incidence of potentially preventable hospitalisations in the first two years of life among children in the National Health Insurance (NHI) system of Taiwan. It also examined income disparities in potentially preventable hospitalisations across four economic categories: below a government-established poverty line and low-, middle-, and upper-income. Five major diseases causing potentially preventable hospitalisations were investigated: gastroenteritis and dehydration, asthma and chronic bronchitis, acute upper respiratory infections, lower respiratory infections, and acute injuries and poisonings.

**Methods:**

NHI data on enrolee registrations and use of ambulatory and hospital care by all children born between July 1, 2003 and June 30, 2004 (n = 218,158) was used for the study. The negative binomial regression method was used to identify factors associated with total inpatient care and the severity level for various types of potentially preventable hospitalisations during the first two years of life.

**Results:**

This study found high inpatient expenses for lower respiratory infections for children in all income categories. Furthermore, results from the multivariate analysis indicate that children in the lowest economic category used inpatient care to a much greater extent than better-off children for problems considered potentially avoidable through primary prevention or through timely outpatient care. This was especially true for acute injuries and poisonings and for lower respiratory infections. On average, and controlling for other variables, a child in poverty spent 6.1 times more days in inpatient care for acute injuries and poisonings (p < 0.01) and 2.7 times more days for lower respiratory infections (p < 0.01) before age two, compared with a similarly-aged high-income child. The results also suggest a connection between economic status and the severity of a condition causing a potentially avoidable hospital admission. On average, length of stay for each admission for gastroenteritis and dehydration for children in poverty was 1.3 times that for high-income children (p < 0.01). Both the ratios for lower respiratory infections and for acute upper respiratory infections were 1.2 (p < 0.01 for both).

**Conclusions:**

There were high hospital admission rates and lengths of stays for lower respiratory infections among young children in all income categories. Hospital care use of young children in the poorest category was significantly higher for acute injuries and poisonings as well as for lower respiratory infections, compared with those of better-off children. The findings suggest the need for increased attention to these two disease types. It particularly calls for more research on the causes of high hospital care use for lower respiratory infections and on the reasons for large economic disparities in hospital care use for acute injuries and poisonings.

## Background

Quality primary and preventive care may reduce the need for hospital care for some health conditions, although it is impossible to eliminate all the need. Over recent years, potentially preventable or avoidable hospitalisations, including ambulatory care-sensitive conditions and those possibly avoidable through primary prevention efforts [1-5], have become a commonly used measure of primary care delivery performance [6,7]. Tracking changes in incidence rates of potentially preventable hospitalisations before and after an intervention has also been a method for evaluating intervention effects [3,8]. A strategy regarded as economical and acceptable for conducting primary investigations of potentially preventable hospitalisations is use of administrative data [4,6,7,9]. For instance, the paediatrics literature has demonstrated use of insurance claims data to examine potentially avoidable hospitalisations in the first two years of life [4].

Investigation of factors associated with potentially avoidable hospitalisations has also been an issue receiving much attention. Factors identified in the literature include preventive care [4,5,10,11], access to hospital care [12,13], healthcare quality [3,14,15], health insurance [8,11,16], continuity with the same care provider [5], educational attainment [17], economic status [8,11,17,18], quasi-economic factors (such as getting time off from work, arranging for child care, and transportation to care sites) [16], location-specific conditions (such as physical accessibility to healthcare) [11,19,20], and environmental factors (such as air quality) [2]. Age, gender, and other demographic characteristics are also identified as factors linked with potentially preventable hospitalisations [11,14,15,21], as is the obvious factor of health status [4,14,15]. According to the literature, almost all factors associated with potentially avoidable hospitalisations affect both adults and children. Concerning these factors, economic disparity in incidence of potentially preventable hospitalisations is a social issue frequently discussed and researched.

To avoid some types of hospitalisations for children under the age of 18, Flores et al. [3] noted that physicians make important contributions via inpatient lay care-taker education and quality outpatient care. Lay care-takers can help prevent hospitalisations by avoiding known disease triggers (such as secondhand smoke at home) and keeping good outpatient follow-up care (including properly administering medications, and obtaining refills before using up medications for children in their care) [3]. The same study identified improvement of housing conditions and advancement of social services as other important ways to reduce potentially preventable hospitalisations. Housing conditions include safety and cleanliness at home, and social services refer to governmental protection against danger and assistance in acquiring necessary healthcare.

As children depend on lay care-takers for seeking healthcare, and epidemiological factors are different for childhood and adult diseases, researchers have developed separate preventable hospitalisation indicators for different age groups [3,4,7,21]. Studies of potentially preventable hospitalisations among children focus on slightly different sets of indicators, reflecting a separation of children into different age categories. For instance, Hakim and Bye [4] investigated potentially preventable hospitalisations for children during the first two years of life, and therefore did not address cellulitis and seizures in the same manner as Parker and Schoendorf [21], who addressed the needs of a much broader age range (1-14 years). Note that Parker and Schoendorf [21] did not examine acute injuries and poisonings in their research.

Although there is an extensive literature on factors associated with potentially avoidable hospitalisations and a body of research on health conditions as indicators of potentially avoidable hospitalisations for different age ranges, some related issues still lack exploration. Two examples are (1) whether the strengths of the links between poverty and total use of inpatient care for potentially preventable hospitalisations differ for different health conditions, and (2) whether poverty is associated with the severity level of a health condition causing a potentially avoidable hospital admission. This present study aims to help fill this knowledge gap by investigating total inpatient care use and severity level for various types of potentially preventable hospitalisations among children from families of different economic classes in Taiwan.

Our investigation focused on the first two years of life. We selected this age range mainly because the paediatrics literature has documented specific health conditions and corresponding disease classification codes that indicate potentially preventable hospitalisations for the two years [4]. The study also examines the link between well-child care use and the incidence of potentially preventable hospitalisations before two years of age in the context of Taiwan because the literature has recommended frequent well-child care visits during the first two years of life and shown a beneficial effect of this healthcare use pattern on reducing potentially avoidable hospitalisations in a U.S. context [4].

The five disease types identified in the literature as indicators of potentially avoidable hospitalisations among children under two are: (1) gastroenteritis and dehydration, (2) asthma and chronic bronchitis, (3) acute upper respiratory infections, (4) lower respiratory infections, and (5) acute injuries and poisonings [4]. We adopted these five disease types as our indicators and used coding of International Classification of Diseases, Ninth Revision, Clinical Modification (ICD-9-CM) to identify them (see Appendix).

Our study chose children enrolled in the National Health Insurance (NHI) system as the sample because the NHI covers almost all Taiwanese children and their medical services and because it maintains a comprehensive database of claims and registration data. The NHI system is a single-payer system with global budgeting. It provides near-universal coverage, free choice of care providers, and equal benefits for all users, regardless of socioeconomic conditions and areas of residence. Households whose incomes are below the national poverty level are exempt from premium and co-payment requirements. While most medical care institutions are private, over 90% of all institutions in Taiwan provide NHI services (including 100% of public medical care institutions). The system has earned a good reputation for reasonable premiums and co-payments, and relatively short wait times for care [22,23]. Data from Taiwan's national surveys also reveal that this system protects almost all Taiwanese and provides medical services in almost all outpatient visits and hospital admissions. For instance, data from the 2005 Taiwan National Health Interview Survey, which collected health-related data from a representative sample of the population of Taiwan, show that 99% of Taiwanese were in the NHI system at the time of interview [24]. The data also indicate that 98% of children under twelve years of age who had outpatient care in the previous month used NHI care on the last visit prior to the interview; 100% of children used NHI services in the last hospital admission prior to the interview among those under twelve years of age who used hospital care in the previous year.

The Taiwan government also provides public well-child care through paediatricians and family medicine practitioners contracting with the NHI. On April 1, 1995, the NHI began providing six well-child care visits for each child in the first four years of life (four in infancy, one in the period covering the second and the third years, and one during the fourth year of life). On July1, 2004, the government expanded the frequency of public well-child care visits. This plan provided nine visits for the first seven years of life: four in infancy, two in the second year, one in the third, one in the fourth, and one in the period covering the fifth to the seventh years of life. However, on January 1, 2010, the government reduced the frequency to seven times: four during infancy, one in the second year, one in the third year, and one in the period covering the fourth to the seventh years of life.

The well-child care services package is the same in rural and non-rural areas, and for various socioeconomic groups. Each well-child care visit includes basic physical examinations following the growth of a child, nutritional consultation, and some education related to primary prevention, such as avoidance of injuries. The government used NHI incomes to pay for the well-child care before 2006, but has been financing these services through general taxes since 2006. Public immunizations (including HBV, OPV, DPT, MV, MMR, JE and BCG), financed through general taxes, can be provided either in well-child care visits or in other outpatient visits.

This study can help distinguish which types of potentially preventable hospitalisations are associated with high poverty-related hazards among young children. It can also help identify other important factors associated with incidence rates and severity levels for various types of potentially avoidable hospitalisations and health conditions with particularly high expenditures on hospital care. The information can be used to advise major scopes of further in-depth studies on reasons causing these potentially preventable hospitalisations and on interventions that may decrease incidences and severity levels of such hospitalisations.

## Methods

We used secondary data from the Taiwan National Health Insurance Research Database, which is provided by the Bureau of National Health Insurance (BNHI) and managed by the National Health Research Institutes (NHRI). This database contains all national health insurance claims and registration data, and is open for research use. The NHRI are responsible for reviewing researchers' applications to use the database and for releasing data to cases meeting their ethical and scientific criteria. We obtained our research data through applications numbered 96027 and 97046.

We used SAS software to construct our analytical data file and administer descriptive analyses. With raw data from the NHI research database, we extracted and organized registration, ambulatory care, and hospital care data for all Taiwanese children with NHI registration data who were born between July 1, 2003 and June 30, 2004. This cohort was selected based on the launch date of the system's most updated program of well-child care before January, 2010. The children in our sample were separated into household economic categories based on existing NHI records indicating the level of family income relative to a poverty line as defined by the BNHI, and the most recent income record of a child's custodian before the child's second birthday, which were used for calculating NHI premiums. We used the data to establish four levels of household income: below the poverty line, low-income (but above the poverty line; referred to as 'near-poverty' hereafter), middle-income, and high-income (Table 1).

**Table 1:** Definitions and distributions of children's economic status

Income level	Definition	All children		Children who experience preventable hospitalisations	
		n	(%)	n	(%)
Poverty	Household income below poverty line as defined by the Bureau of National Health Insurance.	1,829	(0.84)	537	(1.27)
Near poverty	Less than 17,000 NT dollars according to the last record of a custodian's monthly income before a child reaches the age of 2.	54,073	(24.79)	10,595	(25.12)
Middle income	More than 17,001 NT dollars and less than 35,000 NT dollars, according to the last record of a custodian's monthly income before a child reaches the age of 2.	109,382	(50.14)	22,281	(52.82)
High income	35,001 NT dollars or more, according to the last record of a custodian's monthly income before a child reaches the age of 2.	52,874	(24.24)	8,769	(20.79)

1USD = 32 NT dollars as of March 2010.

Other information gathered for this study included NHI registration region before age 2, gender, low birth weight status, number of outpatient visits during the first week of life, and number of well-child care visits during the first two years of life. We removed 0.1% of the children in the original sample due to missing data on household economic status and registration region. Our final sample size was 218,158. These children's characteristics are shown in Table 2. As shown, children in poverty had a higher likelihood of having low birth weight and used significantly less well-child care compared with children in higher economic classes.

**Table 2:** Sample characteristics by economic status

	All children (%)	Poverty (%)	Near poverty (%)	Middle income (%)	High income (%)
*Geographic environment*					
Large city	29.2	22.8	23.7	26.3	41.0
Small city/town	29.7	24.7	28.0	30.4	30.3
Rural area	41.1	52.5	48.3	43.4	28.8
*Physical conditions at birth*					
Gender					
Boy	52.6	53.4	52.7	52.6	52.4
Girl	47.4	46.6	47.3	47.4	47.6
Use of NHI healthcare with low birth weight as a diagnosis					
Yes	2.0	4.0	2.0	1.9	2.1
No	98.0	96.0	98.0	98.1	97.9
Number of outpatient visits during the first week of life					
0	94.9	93.3	94.9	94.7	95.5
1	4.5	6.1	4.4	4.7	4.0
2 or more	0.6	0.6	0.6	0.7	0.5
*Additional physical conditions related to potentially preventable hospitalisations before age two*					
Number of outpatient visits for any of the five disease categories during the first two years of life					
0	1.1	2.1	2.0	0.8	1.0
1-10	9.0	13.2	10.7	7.1	10.8
11-20	18.1	18.4	18.5	16.8	20.5
21-30	20.6	20.1	19.9	20.5	21.5
31 or more	51.2	46.3	48.9	54.8	46.2
*Access to preventive care*					
Number of public well-child care visits used during the first two years of life					
0	5.6	15.1	7.3	4.9	4.8
1-2	14.2	25.9	16.8	13.5	12.4
3-4	28.3	28.1	29.1	28.2	27.9
5-6	51.9	30.9	46.8	53.4	54.9

We conducted the following descriptive analyses for the entire cohort and for each economic group: (1) proportions of children experiencing at least one hospital admission before age two for each of five types of potentially preventable hospitalisations as well as the five disease categories combined; (2) means of "total lengths of stay," "total inpatient care expenses," "the number of admissions," and "the number of outpatient visits" during the first two years of life for each disease type as well as the five disease categories combined. For a child, there could be zero, one, or more than one hospital admission for a specific disease type during the whole period of the first two years of life. For a child with two or more admissions, we added up lengths of stay and inpatient expenditures for all admissions to obtain this child's total length of stay and total expenses. To further understand conditions among children experiencing at least one hospital admission, we also calculated these children's means of total lengths of stay, total inpatient care expenses, the number of admissions, and the number of outpatient visits for each disease type during the first two years of life, as well as the average per-admission expense and length of stay.

We used Stata software for our multivariate analysis. For the entire cohort of children, we investigated factors associated with "total length of stay" for potentially preventable hospitalisations during the first two years of life, separately for each disease type as well as the five disease categories combined. Using the sub-sample of children with at least one potentially avoidable hospital admission, we further examined factors associated with "the duration of an admission," separately for each disease type as well as the five disease categories combined. This was to investigate the severity level of a health condition causing a hospital admission. In the analysis of the total length of stay for the entire first two years of life, each child had exactly one observation. In contrast, a child in the analysis of disease severity might have more than one hospital admission and was hence linked to two or more observations -- one observation here reflected situations for a specific admission, rather than summary conditions for the whole two years.

The Taiwan NHI uses a global budgeting approach, meaning that physicians have less incentive to boost demand for hospital care. Therefore, the primary hospital care demand function in Taiwan is 'need.' Since healthcare needs are primarily determined by health status, important factors in the hospital care demand function are linked to four health determinants: behaviours, environment, biology, and healthcare [25]. Accordingly, for each multivariate model, we included five factors tied to health production: (1) economic status; (2) local environmental conditions; (3) physical conditions at birth; (4) additional physical conditions related to potentially preventable hospitalisations before age two; (5) access to preventive care.

The first and the second factors are associated with behaviours and environment. The third and the fourth mainly reflect biology. The last factor is linked to healthcare. The definition of the first factor was stated previously. We used the urbanization level of NHI registration location to partially capture the influences of local environmental conditions. Physical conditions at birth were reflected by gender, low birth weight status, and the number of outpatient visits during the first week of life. Gender and birth weight are related to physical conditions from the beginning of life [26,27]. The number of outpatient visits during the first week of life also indicates physical conditions at birth to some degree. Additional physical conditions related to potentially preventable hospitalisations before age two can be partially revealed by the numbers of outpatient visits for the entire first two years for the same disease types. Finally, we measured access to preventive care by the number of well-child care visits during the first two years of life. While we were unable to incorporate all potential factors for the hospital care demand function, we used as much information as possible from NHI data to construct a broad set of explanatory factors.

We adopted the negative binomial (NB) regression to estimate these multivariate models. The NB regression is a method commonly used to model the associations of selected covariates with the number of counts of an event (the outcome variable). The process of estimation is based on an NB distribution that can be conceptualised as a mixture of a Poisson distribution and a Gamma distribution [25]. Alternatively, we could use the Poisson regression, which is a special case of NB regression and has one more assumption than the NB method. The assumption is that the conditional mean and the conditional variance of the outcome variable are equal. Another analytical strategy is to measure the number of inpatient days as a continuous variable and adopt ordinary least square (OLS) regression. A fundamental assumption for the OLS method is that the conditional distribution of the residual is a normal distribution.

We estimated each multivariate model using all these three regression methods, and tested fundamental assumptions for the latter two. Our diagnostic results indicate that the assumptions were not satisfied with our data (results available upon request). Therefore, we chose the NB method, which has weaker assumptions. We adopted the Huber/White estimator to acquire robust standard errors and took into account intragroup correlation in each model regarding disease severity linked to an admission [28]. We used incidence-rate ratios (IRR) estimated by the NB models to show the strengths of links of various factors with the amount of hospital care measured in number of inpatient days.

## Results

### Descriptive analysis

Table 3 reports the descriptive analyses for the entire cohort and for each economic group. (We do not report descriptive results for the sub-sample of children who used at least some hospital care.) Slightly under one-fifth (19%) of all the children experienced potentially avoidable hospitalisations before age two. The proportion of children with potentially avoidable hospitalisations among children under the poverty line was 29%, which was 9% higher than the rate for near-poverty and middle-income children, and 12% higher than that for the high-income group.

**Table 3:** Epidemiological data for potentially preventable hospitalisations during the first two years of life by economic status

	All children	Poverty	Near poverty	Middle income	High income
	(n = 218,158)	(n = 1,829)	(n = 54,073)	(n = 109,382)	(n = 52,874)
*Five disease categories combined*					
% of children using hospital care	19.3%	29.4%	19.6%	20.4%	16.6%
Total length of stay (in days; mean)	1.35	4.01	1.46	1.41	1.04
Ratio: length of stay^a^		*3.9*	*1.4*	*1.4*	*ref*.
Total inpatient care expenses (in NT dollars; mean)	5,125.7	16,618.6	5,686.2	5,213.9	3,972.4
Ratio: expense^b^		*4.2*	*1.4*	*1.3*	*ref*.
Number of admissions (mean)	0.3	0.7	0.3	0.3	0.2
Number of outpatient visits (mean)	34.7	32.8	33.5	36.5	32.3
					
*Gastroenteritis and dehydration*					
% of children using hospital care	4.9%	6.9%	5.0%	5.3%	4.1%
Total length of stay (in days; mean)	0.21	0.42	0.22	0.22	0.16
Ratio: length of stay^a^		*2.5*	*1.3*	*1.3*	*ref*.
Total inpatient care expenses (in NT dollars; mean)	627.9	1,337.4	664.2	658.2	503.5
Ratio: expense^b^		*2.7*	*1.3*	*1.3*	*ref*.
Number of admissions (mean)	0.1	0.1	0.1	0.1	0.04
Number of outpatient visits (mean)	2.5	2.2	2.5	2.7	2.4
					
*Asthma and chronic bronchitis*					
% of children using hospital care	0.3%	0.7%	0.3%	0.3%	0.2%
Total length of stay (in days; mean)	0.02	0.04	0.02	0.02	0.01
Ratio: length of stay^a^		*4.0*	*1.8*	*1.6*	*ref*.
Total inpatient care expenses (in NT dollars; mean)	54.9	121.6	67.2	56.8	36.1
Ratio: expense^b^		*3.4*	*1.9*	*1.6*	*ref*.
Number of admissions (mean)	0.003	0.009	0.004	0.003	0.002
Number of outpatient visits (mean)	0.2	0.3	0.2	0.2	0.2
					
					
					
*Acute upper respiratory infections*					
% of children using hospital care	4.2%	4.8%	4.3%	4.5%	3.5%
Total length of stay (in days; mean)	0.17	0.24	0.18	0.18	0.14
Ratio: length of stay^a^		*1.8*	*1.3*	*1.4*	*ref*.
Total inpatient care expenses (in NT dollars; mean)	519.8	706.6	539.5	556.7	416.7
Ratio: expense^b^		*1.7*	*1.3*	*1.3*	*ref*.
Number of admissions (mean)	0.05	0.06	0.05	0.05	0.04
Number of outpatient visits (mean)	25.2	22.7	24.3	26.6	23.5
					
*Lower respiratory infections*					
% of children using hospital care	12.5%	23.5%	12.8%	13.2%	10.5%
Total length of stay (in days; mean)	0.91	3.04	0.99	0.94	0.69
Ratio: length of stay^a^		*4.4*	*1.4*	*1.4*	*ref*.
Total inpatient care expenses (in NT dollars; mean)	3,420.1	11,975.5	3,740.4	3,491.3	2,649.0
Ratio: expense^b^		*4.5*	*1.4*	*1.3*	*ref*.
Number of admissions (mean)	0.2	0.5	0.2	0.2	0.1
Number of outpatient visits (mean)	5.9	6.7	5.7	6.3	5.4
					
*Acute injuries and poisonings*					
% of children using hospital care	0.8%	2.0%	1.0%	0.9%	0.6%
Total length of stay (in days; mean)	0.05	0.27	0.06	0.05	0.04
Ratio: length of stay^a^		*7.4*	*1.7*	*1.3*	*ref*.
Total inpatient care expenses (in NT dollars; mean)	503.1	2,477.4	674.9	451.0	367.0
Ratio: expense^b^		*6.8*	*1.8*	*1.2*	*ref*.
Number of admissions (mean)	0.01	0.02	0.01	0.01	0.01
Number of outpatient visits (mean)	0.8	0.9	0.8	0.8	0.7

1USD = 32 NT dollars as of March 2010.

^a ^This row shows length of stay ratios of the other three economic categories to the high-income category. One digit after the decimal point is reported, which may introduce rounding error.

^b ^This row lists expense ratios of the other three economic categories to the high-income category. One digit after the decimal point is reported, which may introduce rounding error.

The disease type with the highest incidence rate in the whole sample was lower respiratory infections: with a rate of 13%, which was almost 8% higher than the second highest disease type -- gastroenteritis and dehydration. A significant association was found between lower respiratory infections and economic disparity: the means of total length of stay and total expenses for children under poverty were more than 4 times those for children in the wealthiest economic category and more than 3 times those for children in the other two categories.

For the whole sample of children, the proportion of children experiencing hospitalisations for acute injuries and poisonings was the second lowest and only slightly higher than the lowest -- that for asthma and chronic bronchitis. At the same time, the mean length of stay for injuries and poisonings for the entire first two years of life for children in the poverty category was 7.4 times that for the wealthiest economic category, 5.7 times that for middle-income children, and 4.4 times that for near-poverty children. The mean total hospitalisation expenses on injuries and poisonings in the first two years of life for children in poverty was 6.8 times that for children in the wealthiest category, 5.7 times that for middle-income children, and 3.8 times that for near-poverty children.

### Multivariate analysis

Controlling for all other factors, we found a significant association between economic status and total length of stay for potentially preventable hospitalisations for all of the children in our sample (Table 4). The substantially longer total lengths of stay among the poorest children for three disease types warrant special attention: acute injuries and poisonings (IRR = 6.05 relative to high-income children; p < 0.01), lower respiratory infections (IRR = 2.67; p < 0.01), and gastroenteritis and dehydration (IRR = 2.24; p < 0.01). In other words, controlling for all other factors, the mean total length of stay in the first two years of life for acute injuries and poisonings for children from families in poverty was 6.1 times that for children in the high-income category. On average, compared to a high-income child, a child in poverty spent 2.7 times more inpatient days for lower respiratory infections and 2.2 times more inpatient days for gastroenteritis and dehydration during the whole period before two years of age, with all other factors being equal.

**Table 4:** Factors associated with total length of stay for potentially preventable hospitalisations during the first two years of life for all children in the sample: results from the negative binomial regression models

Disease category	All five Diseases			Gastroenteritis and dehydration			Asthma and chronic bronchitis			Acute upper respiratory infections			Lower respiratory infections			Acute injuries and poisonings		
Explanatory variable																		
	**IRR**		**95%CI**	**IRR**		**95%CI**	**IRR**		**95%CI**	**IRR**		**95%CI**	**IRR**		**95%CI**	**IRR**		**95%CI**

*Economic status (reference: high income)*																		
Poverty	2.89	**	2.50-3.34	2.24	**	1.74-2.88	1.83		0.70-4.77	1.70	**	1.31-2.19	2.67	**	2.30-3.10	6.05	**	1.54-23.74
Near poverty	1.26	**	1.19-1.32	1.27	**	1.17-1.37	2.22	**	1.50-3.30	1.16	**	1.08-1.25	1.31	**	1.23-1.39	1.24		0.82-1.89
Middle income	1.17	**	1.12-1.22	1.24	**	1.16-1.33	1.67	*	1.12-2.48	1.22	**	1.14-1.30	1.22	**	1.16-1.29	1.04		0.70-1.53
*Geographic environment (reference: big city)*																		
Small city/town	1.14	**	1.09-1.19	1.12	**	1.05-1.20	1.03		0.67-1.58	1.21	**	1.13-1.29	1.12	**	1.06-1.17	1.20		0.87-1.67
Rural area	1.20	**	1.16-1.26	1.18	**	1.11-1.26	1.03		0.68-1.55	1.36	**	1.28-1.45	1.19	**	1.13-1.26	1.35		0.99-1.84
*Physical conditions at birth*																		
Male	1.20	**	1.16-1.25	1.07	*	1.01-1.12	1.90	**	1.40-2.58	1.18	**	1.12-1.24	1.21	**	1.16-1.26	1.14		0.90-1.44
Low birth weight	2.82	**	2.53-3.13	1.52	**	1.30-1.78	2.88	**	1.36-6.07	1.28	**	1.09-1.52	2.92	**	2.59-3.28	0.53	*	0.29-0.99
Number of outpatient visits in the first week of life	1.17	**	1.11-1.24	1.25	**	1.15-1.36	1.42		0.98.-2.06	1.25	**	1.15-1.36	1.13	**	1.05-1.22	0.78		0.57-1.07
*Additional physical conditions related to potentially preventable hospitalisations before age two*																		
Number of outpatient visits for the same disease before age two	1.03	**	1.03-1.03	1.29	**	1.28-1.30	2.44	**	2.19-2.71	1.02	**	1.02-1.02	1.14	**	1.14-1.15	2.33	**	2.19-2.47
*Access to preventive care*																		
Number of well-child care visits before age two	0.93	**	0.91-0.94	0.98	**	0.96-0.99	0.93		0.86-1.00	1.01		1.00-1.03	0.93	**	0.91-0.95	0.83	**	0.76-0.90

Number of observations	218,158			218,158			218,158			218,158			218,158			218,158		
Model significance: Wald Χ^2^(10)	7,663.18		**	3,501.02		**	301.98		**	1,112.16		**	10,311.12		**	900.49		**

IRR: incidence-rate ratio.

CI: confidence interval.

* p < 0.05; ** p < 0.01.

The positive associations between low birth weight and greater inpatient days during the first two years of life were stronger for lower respiratory infections (IRR = 2.92; p < 0.01) and asthma and chronic bronchitis (IRR = 2.88; p < 0.01) than for the other diseases (Table 4). In contrast, low birth weight was significantly and negatively associated with total length of stay for acute injuries and poisonings (IRR = 0.53; p < 0.05). Results in Table 4 also indicate that the number of outpatient visits and total length of stay during the first two years of life was strongly and positively related for asthma and chronic bronchitis (IRR = 2.44; p < 0.01) and acute injuries and poisonings (IRR = 2.33; p < 0.01).

Higher utilization of well-child care was substantially related to a shorter total inpatient stay in the first two years of life for the disease type with the largest economic disparity. On average, using one more well-child care visit was linked to a 17% reduction of total inpatient stay for acute injuries and poisoning (IRR = 0.83; p < 0.01). This also implies that a child using six well-child care visits could have a 67% reduction of total inpatient stay for acute injuries and poisoning (= 1 - 0.83^6^) compared to a child who did not use well-child care before two years of age. The association between greater use of well-child care and less hospital care was also noteworthy for lower respiratory infections (IRR = 0.93; p < 0.01). This finding suggests that a child using six well-child care visits could have a 35% reduction in total inpatient stays for lower respiratory infections (= 1 - 0.93^6^) compared with a child that had no well-child care visit.

Controlling for all other factors, economic status also had a substantial association with per-admission length of stay among children experiencing potentially preventable hospitalisations. This suggests a connection between economic status and condition severity causing hospital care utilisation (Table 5). On average, per-admission length of stay for gastroenteritis and dehydration for children in poverty was 1.3 times that for high-income children (p < 0.01). Both the ratios for lower respiratory infections and for acute upper respiratory infections were 1.2 (p < 0.01 for both). Although it was not statistically significant at the level of p = 0.05 (partly due to a smaller sample size), the ratio for acute injuries and poisonings (IRR = 1.82; the 95% confidence interval (CI): 0.94~3.53) warrants more research.

**Table 5:** Factors associated with length of stay per admission among children experiencing potentially preventable hospitalisations during the first two years of life: results from the negative binomial regression models

Disease category	All five Diseases			Gastroenteritis and dehydration			Asthma and chronic bronchitis			Acute upper respiratory infections			Lower respiratory infections			Acute injuries and poisonings		
Explanatory variable																		
	**IRR**		**95%CI**	**IRR**		**95%CI**	**IRR**		**95%CI**	**IRR**		**95%CI**	**IRR**		**95%CI**	**IRR**		**95%CI**

*Economic status (reference: high income)*																		
Poverty	1.26	**	1.18-1.36	1.25	**	1.10-1.41	0.99		0.73-1.33	1.19	**	1.05-1.36	1.18	**	1.10-1.26	1.82		0.94-3.53
Near poverty	1.04	**	1.02-1.07	1.04	*	1.01-1.08	1.13		1.00-1.27	1.02		0.98-1.05	1.04	**	1.01-1.07	0.92		0.75-1.13
Middle income	1.01		0.99-1.03	1.01		0.98-1.04	1.12	*	1.00-1.26	1.02		0.99-1.05	1.02		0.99-1.04	0.80	*	0.66-0.96
*Geographic environment (reference: big city)*																		
Small city/town	1.00		0.98-1.02	0.99		0.96-1.02	1.02		0.88-1.19	1.01		0.98-1.04	1.00		0.98-1.02	1.04		0.88-1.21
Rural area	1.00		0.98-1.02	1.00		0.97-1.03	0.91		0.80-1.03	1.00		0.97-1.03	1.00		0.97-1.03	0.99		0.86-1.14
*Physical conditions at birth*																		
Male	1.03	**	1.01-1.05	1.01		0.98-1.03	0.98		0.87-1.10	1.00		0.98-1.02	1.03	**	1.01-1.05	0.99		0.88-1.11
Low birth weight	1.27	**	1.21-1.33	1.12	**	1.03-1.21	1.29	*	1.01-1.65	1.05		0.98-1.14	1.25	**	1.18-1.31	0.82		0.60-1.13
Number of outpatient visits in the first week of life	1.00		0.98-1.03	1.01		0.97-1.05	1.06		0.91-1.23	1.03		1.00-1.06	1.00		0.97-1.04	0.93		0.79-1.09
*Additional physical conditions related to potentially preventable hospitalisations before age two*																		
Number of outpatient visits for the same disease before age two	1.00	**	1.00-1.00	1.01	**	1.00-1.01	1.01	**	1.00-1.02	1.00	**	1.00-1.00	1.00	**	1.00-1.00	1.05	**	1.04-1.06
*Access to preventive care*																		
Number of well-child care visits before age two	0.97	**	0.96-0.98	0.98	**	0.97-0.99	0.99		0.96-1.01	0.99		0.99-1.00	0.97	**	0.96-0.98	0.93	**	0.90-0.97

Number of observations	63,796			11,896			685			10,154			39,146			1,915		
Number of clusters	42,182			10,752			600			9,219			27,283			1,840		
Model significance: Wald Χ^2^(10)	452.77		**	76.99		**	25.10		**	38.57		**	393.17		**	116.46		**

IRR: incidence-rate ratio.

CI: confidence interval.

* p < 0.05; ** p < 0.01.

Similar to findings on total length of stay during the first two years of life, low birth weight had a stronger association with a longer per-admission length of stay for lower respiratory infections (IRR = 1.25; p < 0.01) and asthma and chronic bronchitis (IRR = 1.29; p < 0.01) than for the other disease types. It is noteworthy that having one more well-child care visit was also linked to a 7% reduction in per-admission length of stay for acute injuries and poisoning (IRR = 0.93; p < 0.01). This suggests that a child using six well-child care visits could have a 35% reduction of episode severity for this disease type (= 1 - 0.93^6^) compared with a child having no well-child care visit before two years of age.

## Discussion

Taiwanese per capita health expenditures in 2006 were 31,661 New Taiwan dollars (NT dollars) [29]. A comparison of this amount with expenditures for potentially preventable hospitalisations in the first two years of life highlights the amount of resources spent on young children's hospital care that may be avoided or reduced by adequate and timely ambulatory care as well as primary prevention. Three findings from this study are particularly noteworthy: (1) high hospital admission rates and lengths of stays for lower respiratory infections among young children in all income categories, (2) significantly higher hospital care use among young children in the poorest category for acute injuries and poisonings as well as for lower respiratory infections, and (3) substantial links of more use of well-child care with a shorter total length of stay and with lower episode severity for acute injuries and poisonings in the first two years of life. We have not found any published article that reports information on how poverty and well-child care are associated with specific types of potentially preventable hospitalisations among children. These findings thus provide new information for related fields.

Behavioural and environmental factors may be two reasons for economic inequalities in incidences of potentially preventable hospitalisations, as the literature shows the correlations of poverty with unhealthy behaviours and detrimental environments [30-32]. According to the U.S. Department of Health and Human Services (DHHS) [33], poor indoor air quality (such as that due to use of tobacco indoors) is a disease trigger for pneumonia, bronchitis, and asthma, especially for the first two years of life. The U.S. DHHS also notes that low-income adults tend to smoke more and are less likely to purposefully avoid smoking around their children. Similarly, according to the 2005 Taiwan National Health Interview Survey, 9.2% experienced coughs and 1.4% experienced tachypnoea induced by secondhand smoke among children who were in the lowest household income category defined by this report, while only 7.1% experienced coughs and 0.5% experienced tachypnoea induced by secondhand smoke among better-off children [24]. This may explain, at least in part, the economic disparity we observed in hospitalisations due to respiratory diseases. A similar association has been found between economic status and child safety [34,35], which partly explains why poor children suffer from higher incidences of acute injuries and poisonings.

Regarding healthcare access, while direct cost of healthcare can obstruct poor people from acquiring timely treatment, quasi-economic factors (such as getting time off from work, obtaining child care, and transportation to healthcare facilities) are also important factors that prevent children in low-income households from receiving all necessary health services [16]. These same factors may also explain, at least in part, the economic inequalities in potentially avoidable hospitalisations that are still observed among young Taiwanese children despite the fact that there are no or low direct monetary barriers to healthcare in this country. As shown in the report of findings from the 2005 Taiwan National Health Interview Survey, 12.5% of children who were in the lowest household income category defined by this report and did not receive medical care for an illness in the previous year failed to seek care because their parents or lay care-takers did not have time to bring them to get care [24]. In contrast, the corresponding rate for the second lowest income category was 4.5%, and that for the highest income category was 1.7%. The same report also indicates that only 0.1% of children failed to get medical care mainly due to lack of NHI support among those who had to give up seeking care for illness sometimes in the previous year, and 92.0% did not get care because their parents or lay care-takers thought that the condition was very minor and needed no care from healthcare facilities.

Dafny and Gruber [12] noted that providing public health insurance and/or services and increasing healthcare efficiency are common strategies for reducing potentially preventable hospitalisations. However, such policies cannot eliminate disadvantages due to quasi-economic factors and disease triggers that include living environments and parents' or lay care-takers' behaviours. Our data were insufficient for examining whether the NHI has mitigated differences in potentially preventable hospitalisations among young children from different economic backgrounds since our data only show situations after the NHI was introduced. Kaestner et al. [18] used a difference-in-differences approach, and failed to demonstrate any evidence that the 1988-1992 expansion in the U.S. Medicaid program reduced healthcare disparities of potentially avoidable hospitalisations due to economic inequality among children two years old or younger. It should be noted that Medicaid and private insurance programs in the U.S. have different payment schemes and that children in different programs may receive care at very different quality levels [18]. In Taiwan, NHI physicians participate in the same payment scheme regardless of their patients' socioeconomic conditions, so there is no overt incentive for them to favour patients in more affluent economic categories. Therefore, this NHI may have exerted a more positive effect on decreasing economic inequality in children's potentially preventable hospitalisations compared to the U.S. Medicaid expansion program in the early 1990s.

In the meantime, more effort may be necessary to increase efficiency in the NHI system and further decrease such inequality. Although it is not expected that NHI can remove all healthcare disparities, enhancing quality of primary care (including well-child care) in the NHI system may help further reduce economic inequality in children's potentially preventable hospitalisations, as well as total expenditures. Intervention efforts aimed at reducing potentially preventable hospitalisations need to focus on high-risk areas [2]. According to the theory of concave health production functions, public investments in reducing potentially preventable hospitalisations among poor citizens have the potential to yield Pareto improvement in health throughout a society. In other words, improving the health of poor families can be accomplished without affecting the health of more economically secure citizens and without using more social resources [2].

One tactic for reducing avoidable hospitalisations for children involves educational sessions and counselling services for parents and lay care-takers, with a focus on protecting children from diseases and finding healthcare assistance when they do get sick. Evidence from Janson [36] and Ralston and Roohi [37] indicates that counselling and information sharing sessions when children are hospitalised can exert positive influences on parents' and lay care-takers' subsequent behaviours. Information on general health and prevention can be disseminated at these sessions along with knowledge on direct and indirect costs to families due to potentially avoidable hospitalisations as a means of encouraging positive behavioural change, especially among parents and lay care-takers in low-income families.

In Taiwan, as well as other countries providing public well-child care, information can also be disseminated as part of the national well-child care services program, as findings from this study indicate that greater use of well-child care was substantially associated with a shorter total length of stay for potentially preventable hospitalisations before age two and with lower episode severity of hospitalisation for some diseases. Concerning this respect, it is a pity that the Taiwan government has reduced the frequency of public well-child care visits to seven times from nine times since January 1, 2010. The reduction includes one visit before age two. Reasons for this policy change are not clear, but serious financial pressure for the government in a poor economy and lack of confidence in the benefits of providing well-child care could explain the cuts. While this study can not provide evidence on causal effects of well-child care use on future healthcare savings through the avoidance of hospital care, findings from this study suggest that it is very likely to be worthwhile to invest in providing well-child care. Therefore, we strongly recommend that the government support in-depth research on whether and how well-child care services can reduce later healthcare cost. Such research should include a strong qualitative component to unearth services that have the potential to reduce future sickness or harm, as well as a subsequent intervention program with treatment and control groups to confirm findings from the previous qualitative research. Information from such research can help the government in designing future policies and interventions.

Policy makers also need to consider social support for poor children in forms other than free healthcare as opportunity costs other than direct healthcare costs are also important barriers to healthcare for poor families. Since the NHI expenses for potentially preventable hospitalisations are significant and particularly high for children in poverty, it is necessary to establish a range of interventions for the welfare of children. For instance, the government can launch assistance programs to encourage parents or other lay care-takers in poor families with children to bring their children to obtain well-child care and necessary medical care for illness. The forms of help could include the provision of free transportation to poor families for getting care, social works to escort poor children to seek care when their lay care-takers are too busy to do this, and physicians and public nurses to deliver primary healthcare in poor villages.

In reality, not all potentially preventable hospitalisations can be avoided. Despite this, findings from this study suggest that we should pay more attention to two disease types: "acute injuries and poisonings" and "lower respiratory infections." The findings particularly call for more research on the causes of high hospital care use for lower respiratory infections and on the reasons for large economic disparities in hospital care use for acute injuries and poisonings. Both qualitative research and intervention programs that include thoughtful post-intervention evaluations should be conducted in order to identify strategies to reduce healthcare costs through better primary prevention and more efficient ambulatory care use.

Three variable-related caveats should be mentioned. First, although we could precisely identify children under poverty using NHI data, economic status for the other three income categories is measured with error. This is because income level is only observed for a child's custodian who registered the child in the NHI system. This custodian is either the only income earner of the family or the one with lower income of both parents having incomes. Further, the data do not allow us to observe total family income or family size. This limitation did not prohibit us from identifying types of potentially preventable hospitalisations with high expenditures for the whole sample of children. Furthermore, with this limitation, we could still derive reliable conclusions regarding diseases for which poor children had higher risk of potentially preventable hospitalisations.

Second, NHI registers families according to the location of the primary caregiver's employer or institute (including local government) responsible for providing NHI registration information. Therefore, urbanization level does not fully reflect the locations where children actually reside before age two. Third, we identified children with low birth weight by using NHI data on whether a child received any NHI healthcare with low birth weight as a diagnosis. Accordingly, it is likely that this study only identified children with very low birth weight, and the low birth weight incidences were underestimated.

## Conclusions

Three findings from this study are particularly noteworthy: (1) there are high hospital admission rates and lengths of stays for lower respiratory infections among young children in all income categories, (2) there is significantly higher hospital care use among young children in the poorest category for acute injuries and poisonings as well as for lower respiratory infections, and (3) there are substantial links between greater use of well-child care and a shorter total length of stay and with lower episode severity for acute injuries and poisonings in the first two years of life. These findings suggest that more attention should be paid to two disease types: acute injuries and poisonings as well as lower respiratory infections. Particularly, it calls for more research on the causes of high hospital care use for lower respiratory infections and on the reasons for large economic disparities in hospital care use for acute injuries and poisonings. It is also worthwhile to conduct additional research on whether and how well-child care services can reduce later healthcare costs.

## Competing interests

The authors declare that they have no competing interests.

## Authors' contributions

LC designed the study, participated in data analysis, and took primary responsibility for drafting the manuscript. H-ML participated in designing the study and writing the manuscript, and took primary responsibility for analysing data. S-FS, KNK, C-LC and LCH participated in interpreting results and writing the manuscript. All authors have read and approved the manuscript.

## Appendix

### Coding for the five disease types based on the International Classification of Diseases, Ninth Revision, Clinical Modification (ICD-9-CM)

1. Gastroenteritis and dehydration: 009, 276.0, 276.1, 276.5, 558.9, 775.5

2. Asthma and chronic bronchitis: 491.21, 491.9, 493

3. Acute upper respiratory infections: 460-465

4. Lower respiratory infections: 466, 481-483, 485-486

5. Acute injuries and poisonings: 521.1, 360.5-360.6, 376.6, 388.11, 800-994.9 (excluding 855-859,888-889, 898), 995.5-995.9, E800-E928.9, E950-E958.9, E960-E968.9, E980-E999, V71.4, V71.6

## Pre-publication history

The pre-publication history for this paper can be accessed here:

http://www.biomedcentral.com/1472-6963/10/196/prepub

## References

[B1] Caminal J Starfield B Sánchez E Casanova C Morales M The role of primary care in preventing ambulatory care sensitive conditions Eur J Public Health 2004 14 3 246 251 10.1093/eurpub/14.3.246 15369028

[B2] Delia D Distributional issues in the analysis of potentially preventable hospitalisations Health Serv Res 2003 38 6p2 1761 1780 10.1111/j.1475-6773.2003.00201.x 14727796 PMC1360972

[B3] Flores G Abreu M Chaisson CE Sun D Keeping children out of hospitals: parents' and physicians' perspectives on how pediatric hospitalisations for ambulatory care-sensitive conditions can be avoided Pediatrics 2003 112 5 1021 1030 10.1542/peds.112.5.1021 14595041

[B4] Hakim RB Bye BV Effectiveness of compliance with pediatric preventive care guidelines among Medicaid beneficiaries Pediatrics 2001 108 1 90 97 10.1542/peds.108.1.90 11433059

[B5] Kozak LJ Hall MJ Owings MF Trends in avoidable hospitalisations, 1980-1998 Health Aff 2001 20 2 225 232 10.1377/hlthaff.20.2.225 11260947

[B6] Prevention quality indicators overview. AHRQ quality indicators 2004 http://www.qualityindicators.ahrq.gov/pqi_overview.htm

[B7] Pediatric quality indicators overview. AHRQ quality indicators 2006 http://www.qualityindicators.ahrq.gov/pqi_overview.htm

[B8] Laditka SB Laditka JN Geographic variation in preventable hospitalisation of older women and men: implications for access to primary health care J Women Aging 1999 11 4 43 56 10.1300/J074v11n04_04 10721688

[B9] Basu J Friedman B Burstin H Managed care and preventable hospitalisation among Medicaid adults Health Serv Res 2004 39 3 489 510 10.1111/j.1475-6773.2004.00241.x 15149475 PMC1361021

[B10] Gadomski A Jenkins P Nichols M Impact of a Medicaid primary care provider and preventive care on pediatric hospitalisation Pediatrics 1998 101 3 e1 10.1542/peds.101.3.e1 9481020

[B11] Shi L Samuels ME Pease M Bailey WP Corley EH Patient characteristics associated with hospitalisations for ambulatory care sensitive conditions in South Carolina South Med J 1999 92 10 989 998 10548172 10.1097/00007611-199910000-00009

[B12] Dafny L Gruber J Public insurance and child hospitalisations: access and efficiency effects J Public Econ 2005 89 1 109 129 10.1016/j.jpubeco.2003.05.004

[B13] Saha S Solotaroff R Oster A Bindman AB Are potentially preventable hospitalisations sensitive to changes in access to primary care? The case of the Oregon Health Plan Med Care 2007 45 8 712 719 10.1097/MLR.0b013e318053717c 17667304

[B14] O'Malley AS Pham HH Schrag D Wu B Bach PB Potentially avoidable hospitalisations for COPD and pneumonia: the role of physician and practice characteristics Med Care 2007 45 6 562 570 10.1097/MLR.0b013e3180408df8 17515784

[B15] Rizza P Bianco A Pavia M Angelillo IF Preventable hospitalisation and access to primary healthcare in an area of Southern Italy BMC Health Serv Res 2007 7 134 10.1186/1472-6963-7-134 17760976 PMC2045098

[B16] Billings J Anderson GM Newman LS Recent findings on potentially preventable hospitalisations Health Aff 1996 15 3 239 249 10.1377/hlthaff.15.3.239 8854530

[B17] Blustein J Hanson K Shea S Potentially preventable hospitalisations and socioeconomic status Health Aff 1998 17 2 177 189 10.1377/hlthaff.17.2.177 9558796

[B18] Kaestner R Racine A Joyce T Did recent expansions in Medicaid narrow socioeconomic differences in hospitalisation rates of infants? Med Care 2000 38 2 195 206 10.1097/00005650-200002000-00009 10659693

[B19] Parchman ML Culler SD Potentially preventable hospitalisations in primary care shortage areas. An analysis of vulnerable Medicare beneficiaries Arch Fam Med 1999 8 6 487 491 10.1001/archfami.8.6.487 10575386

[B20] Zhang W Mueller KJ Chen LW Uninsured hospitalisations: rural and urban differences J Rural Health 2008 24 2 194 202 10.1111/j.1748-0361.2008.00158.x 18397456

[B21] Parker JD Schoendorf KC Variation in hospital charges for ambulatory care-sensitive conditions among children Pediatrics 2000 106 4 942 948 11044148

[B22] Lu JR Hsiao WC Does universal health insurance make healthcare unaffordable? Lessons from Taiwan Health Aff 2003 22 77 88 10.1377/hlthaff.22.3.77 12757274

[B23] Reinhardt U Humbled in Taiwan Br Med J 2008 336 72 10.1136/bmj.39450.473380.0F PMC2190287 18187722

[B24] Report of findings from the 2005 Taiwan National Health Interview Survey, No. 1 http://nhis.nhri.org.tw/files/2005NHIS_Final%20Report_1.pdf

[B25] Institute of Medicine Improving health in the community 1997 Washington, DC: National Academy Press

[B26] Ghosh R Rankin J Pless-Mulloli T Glinianaia S Does the effect of air pollution on pregnancy outcomes differ by gender? A systematic review Environ Res 2007 105 3 400 408 10.1016/j.envres.2007.03.009 17493608

[B27] Phillips DIW Programming of the stress response: a fundamental mechanism underlying the long-term effects of the fetal environment? J Intern Med 2007 261 5 453 460 10.1111/j.1365-2796.2007.01801.x 17444884

[B28] Stata Corporation Stata User's Guide Release 7 2001 College Station: Stata Press

[B29] Health Statistics 2007 http://www.doh.gov.tw/CHT2006/DM/DM2_2.aspx?now_fod_list_no=10251&class_no=440&level_no=1

[B30] Case A Lubotsky D Paxson C Economic status and health in childhood: the origins of the gradient Am Econ Rev 2002 92 5 1308 1334 10.1257/000282802762024520 29058397

[B31] Denton M Walters V Gender differences in structural and behavioural determinants of health: an analysis of the social production of health Soc Sci Med 1999 48 1221 1235 10.1016/S0277-9536(98)00421-3 10220021

[B32] Stronks K van de Mheen HD Looman CWN Machenbach JP Behavioral and structural factors in the explanation of socio-economic inequalities in health: an empirical analysis Sociol Health Ill 1996 18 5 653 674 10.1111/1467-9566.ep10934524

[B33] U.S Department of Health and Human Service Children and Secondhand Smoke Exposure. Excerpts from The Health Consequences of Involuntary Exposure to Tobacco Smoke: A Report of Surgeon General 2007 Atlanta: U.S. Department of Health and Human Service, Centers for Disease Control and Prevention, Coordinating Center for Health Promotion, National Center for Chronic Disease Prevention and Health Promotion, Office on Smoking and Health

[B34] Hurtado AM Lambourne CA Hill KR Kessler K The public health implications of maternal care trade-offs Hum Nat 2006 17 2 129 154 10.1007/s12110-006-1014-y 26181411

[B35] Rhodes KV Iwashyna TJ Child injury risks are close to home: parent psychosocial factors associated with child safety Matern Child Health J 2007 11 3 269 275 10.1007/s10995-006-0171-2 17216351

[B36] Janson C The effect of passive smoking on respiratory health in children and adults Int J Tuberc Lung Dis 2004 8 5 510 516 15137524

[B37] Ralston S Roohi M A randomized, controlled trial of smoking cessation counselling provided during child hospitalisation for respiratory illness Pediatr Pulmonol 2008 43 561 566 10.1002/ppul.20810 18433044

